# Editorial: Design, Synthesis and Biomedical Applications of Functional Polymers

**DOI:** 10.3389/fchem.2021.681189

**Published:** 2021-07-01

**Authors:** Changkui Fu, Chongyu Zhu, Christopher V. Synatschke, Xiaoyong Zhang

**Affiliations:** ^1^Australian Institute for Bioengineering and Nanotechnology, The University of Queensland, Brisbane, QLD, Australia; ^2^Department of Materials Science, Fudan University, Shanghai, China; ^3^Max Planck Institute for Polymer Research, Mainz, Germany; ^4^Department of Chemistry, Nanchang University, Nanchang, China

**Keywords:** biomedical application, functional polymers, synthesis, design, stimuli-response materials, biodearadable polymer

With the advancement of synthetic polymer chemistry, researchers are now able to readily prepare polymers with tailored composition, architecture, and functionality. This has greatly expanded the application of polymers in many areas. In particular, functional polymers as soft and biocompatible materials have been more and more involved in various biomedical applications. Facilitated by elegantly-designed functional polymers, tremendous progress has been achieved in the biomedical field including but not limited to the development of stimuli-responsive polymer materials, innovative drug delivery systems, and advanced theranostics. Chemical design and synthesis of polymers undoubtedly plays an important role in driving these achievements. On the other hand, for practical biomedical applications, polymers need to meet many requirements. Among those biocompatibility and biodegradability of polymers are profoundly necessary properties. Synthetic technologies leading to biocompatible and biodegradable polymers such as polyesters, polycarbonates and poly(amino acids) are therefore of great interest. The goal of this Research Topic is to highlight recent developments in synthetic polymer chemistry and functional polymers for biomedical applications. Below we highlight 5 papers included in this Research Topic on the synthesis and biomedical application of stimuli-responsive polymers and biocompatible/biodegradable polymers.

Stimuli-responsive polymeric materials have attracted considerable interest due to their ability to respond to specific physical, chemical or biological triggers. This character is particularly useful for targeted drug delivery and imaging of pathological changes in diseased tissue. In the original research article, Zhao et al. reported the synthesis of poly(ethylene glycol) (PEG) with a single cinnamaldehyde acetal unit in the polymer chain. The incorporation of cinnamaldehyde acetal unit makes the polymer pH-responsive. In acidic environment, the acetal linkage would be hydrolyzed and the polymer can be degraded into PEG fragments and release cinnamaldehyde, a bioactive molecule. They further used this polymer to prepare a whole PEG-based hydrogel via thiol-ene reaction. The hydrogel can be completely degraded under low pH condition, demonstrating potential as a smart drug delivery system for therapeutic application. Intracellular delivery enables more efficient drug delivery and therapeutic efficacy but is a critical and challenging process. By utilizing functional polymers and polymer-associated nanoparticles, a number of effective approaches have been developed for intracellular delivery applications. In a mini-review, Wang summarized the design and application of pH-responsive amphiphilic carboxylate polymers as innovative drug delivery system to transport therapeutic payloads across cell membrane and release them in endosomes. The mini-review also provides mechanistic insights into the effect of different carboxylate polymers on their endosomal escape properties, and highlights the challenges and future opportunities for this kind of polymers for intracellular delivery applications.

Polyesters and polycarbonates are important class of biocompatible and biodegradable polymers supporting a myriad of biomedical applications. Synthetic methodologies that are able to produce these polymers from readily-available chemical building blocks in an efficient and green manner are highly needed. In a review article, Liang et al. summarized recent developments in ring-opening copolymerization (ROCOP) of epoxides with carbon dioxide and cyclic anhydrides to prepare polyesters and polycarbonates. Specifically, they highlighted the advances in the catalysts used in ROCOP including organometallic complexes and metal-free Lewis pairs systems. They also reviewed the progress of using post-polymerization modification strategy to prepare polyesters and polycarbonates with functional groups such as hydroxyl and alkene groups that can be used as chemical handles for further functionalization. In addition to polyesters and polycarbonates, poly(amino acids) represent another important class of biocompatible and biodegradable polymers that demonstrate a broad range of biomedical applications. In a mini-review, Chen et al. summarized the development of the synthesis, modification and biomedical applications of epsilon-poly-L-lysine and L-lysine-based dendrimers. They reviewed the diverse applications of these lysine-based polymers as effective antimicrobial and antiviral agents, functional adjuvants, and innovative drug delivery systems. An effective approach leading to poly(amino acids) is ring opening polymerization of cyclic monomers of N-(thio)carboxyanhydrides (N(T)CA. Due to the lack of effective experimental tools, a clear understanding of the reaction mechanism and kinetics in ROP of N(T)CA remains challenging. This however can be realized by the assistance of advanced computational chemistry. In a mini-review, Bai et al. summarized the use of density functional theory (DFT) calculations to investigate the reaction details and overall kinetics of ROP of N(T)CA, and to optimize the structure of monomers, initiators and catalysts for the polymerization. This article will be very useful for synthetic polymer chemists to gain deeper insight into the reaction mechanism of ROP of N(T)CA at molecular level.

## Author Contributions

CF wrote the editorial. CZ, CS, and XZ reviewed the editorial. All authors contributed to the article and approved the submitted version.

## Conflict of Interest

The authors declare that the research was conducted in the absence of any commercial or financial relationships that could be construed as a potential conflict of interest.

